# Supplementary material for: Effects of Eugenol on Water Quality and the Metabolism and Antioxidant Capacity of Juvenile Greater Amberjack (Seriola dumerili) under Simulated Transport Conditions
Source: Animals (Basel). 2022 Oct 21;12(20):2880. doi: 10.3390/ani12202880 (PMC9598218; doi:10.3390/ani12202880)
Supplement: Supplementary file 1 [file animals-12-02880-s001.zip › animals-1935027-supplementary.pdf]

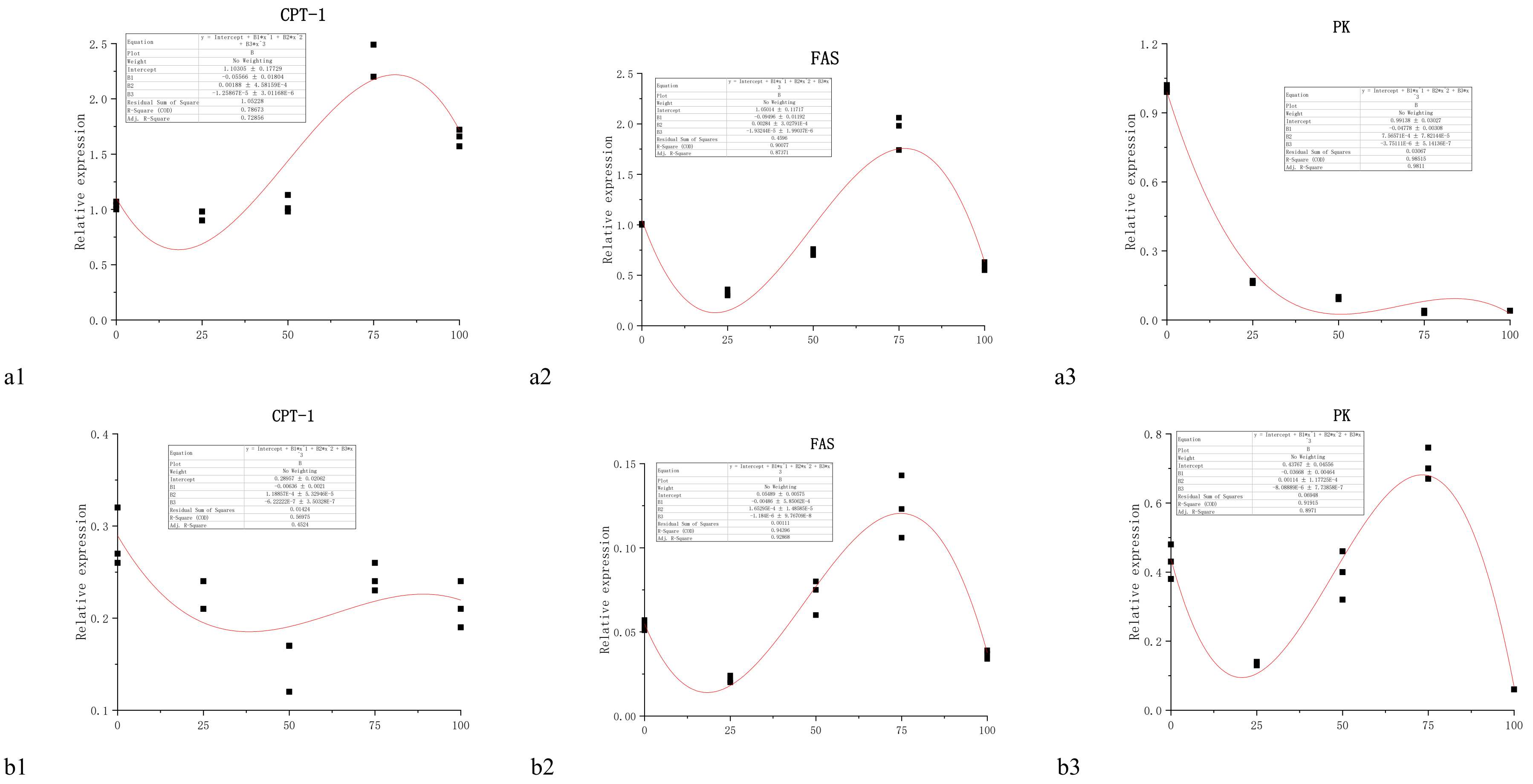

Figure S1. (I) Linear regression equations between eugenol and the relative expression of CPT-1, FAS, and PK in liver (a1, a2, a3) and gill (b1, b2, b3) tissues of the greater amberjack.

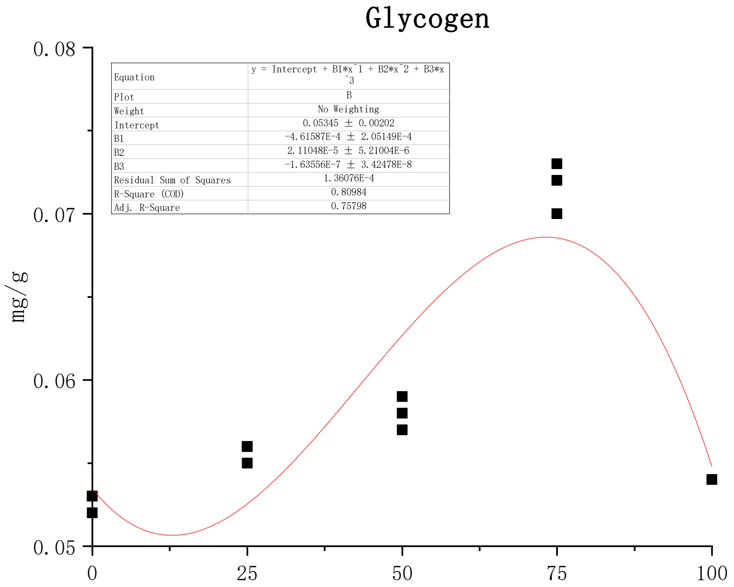

a

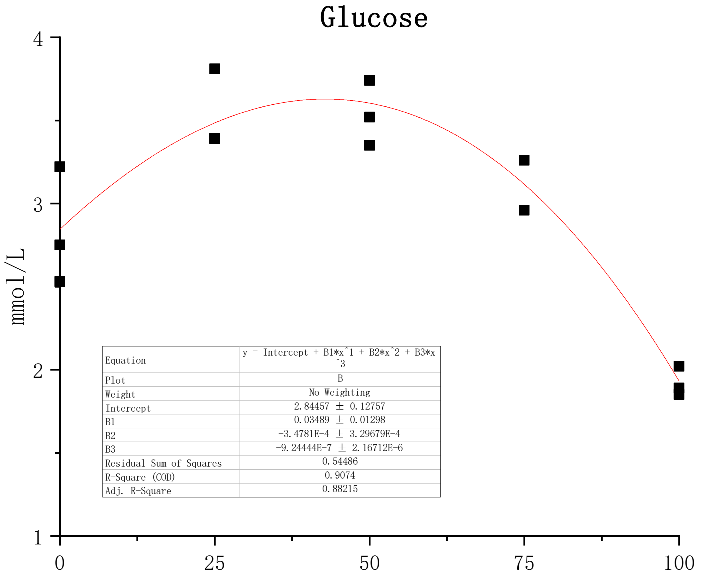

b

Figure S1. (II) Linear regression equations of eugenol with liver glycogen (a) and serum glucose (b) concentrations in greater amberjack under simulated transport conditions.

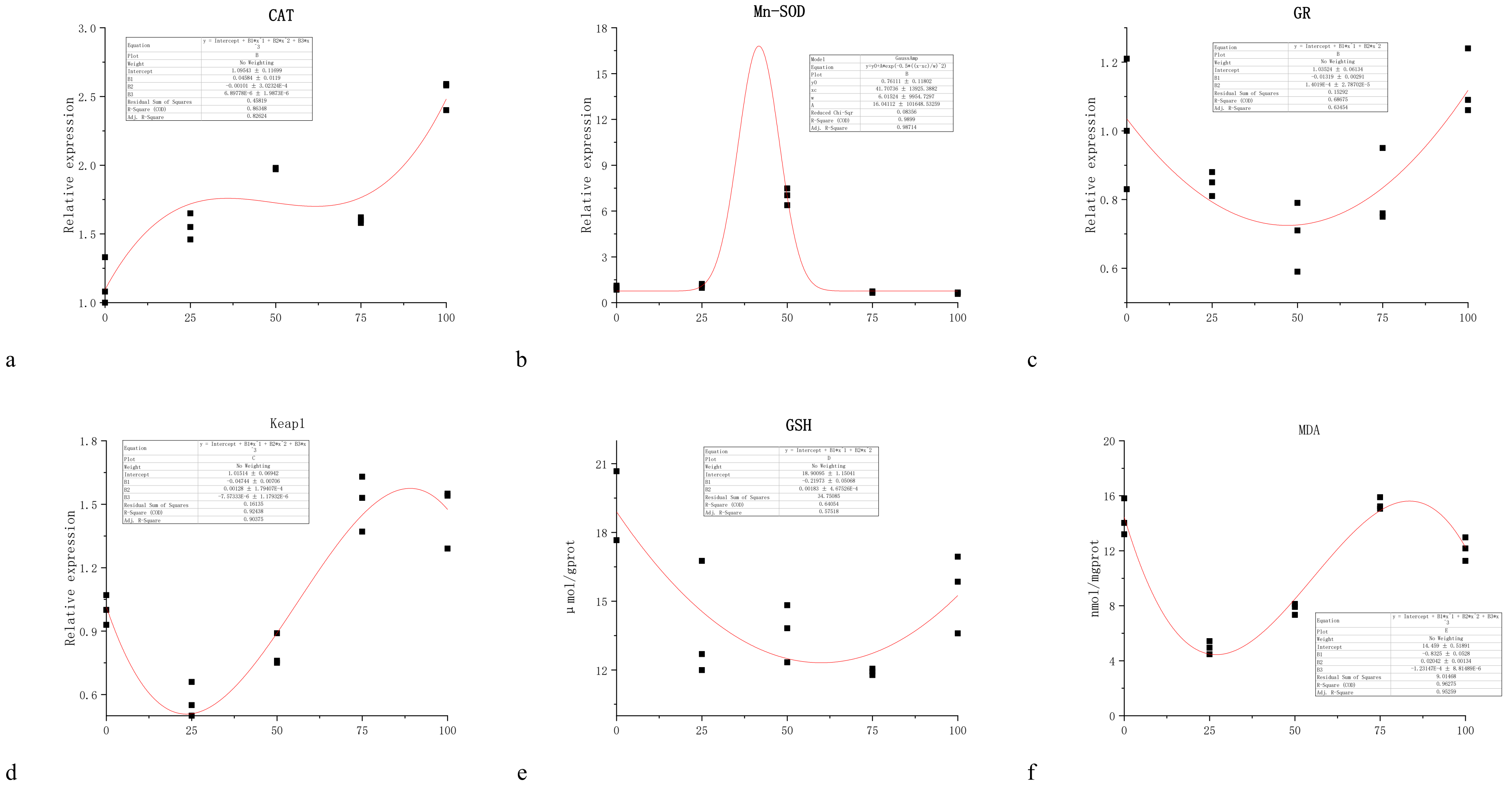

Figure S1. (III) Linear regression equations of eugenol on antioxidant-related genes (a: CAT; b: Mn-SOD; c: GR; d: Keap1) and antioxidant-related enzymes (e: GSH; f: MDA) in the liver of the greater amberjack under simulated transport conditions.

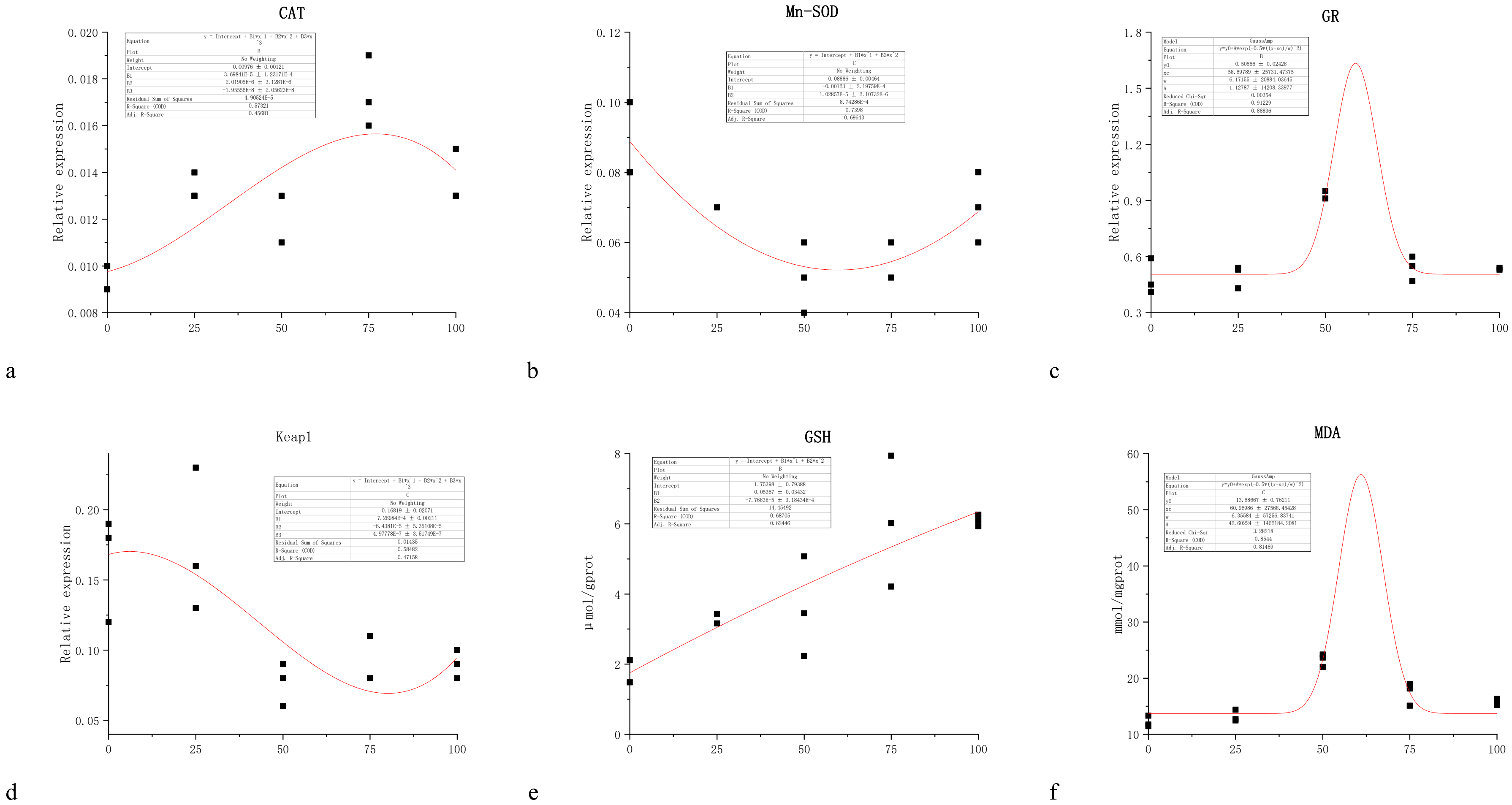

Figure S1. (IV) Linear regression equations of eugenol on antioxidant-related genes (a: CAT; b: Mn-SOD; c: GR; d: Keap1) and antioxidant-related enzymes (e: GSH; f: MDA) in the gill of the greater amberjack under simulated transport conditions.
